# Identifying Health-Related Conditions Associated with Tinnitus in Young Adults

**DOI:** 10.3390/audiolres13040048

**Published:** 2023-07-20

**Authors:** Ishan Sunilkumar Bhatt, Nilesh J. Washnik, Sarah Kingsbury, Aniruddha K. Deshpande, Hailey Kingsbury, Srividya Grama Bhagavan, Klayre Michel, Raquel Dias, Ali Torkamani

**Affiliations:** 1 Department of Communication Sciences and Disorders, University of Iowa, Iowa City, IA 52242, USA; 2 Department of Hearing Speech and Language Sciences, Ohio University, Athens, OH 45701, USA; 3 Department of Speech-Language-Hearing Sciences, Hofstra University, Hempstead, NY 11549, USA; aniruddha.deshpande@hofstra.edu; 4 Department of Microbiology and Cell Science, University of Florida, Gainesville, FL 32603, USA; 5 Department of Integrative Structural and Computational Biology, Scripps Science Institute, La Jolla, CA 92037, USA

**Keywords:** tinnitus, epidemiology, cochlear synaptopathy, noise, COVID-19, prevalence, risk factor, tinnitus phenotypes

## Abstract

Objective: The present study investigated the epidemic of tinnitus in college-aged young adults. Our first objective was to identify health conditions associated with tinnitus in young adults. The second objective was to evaluate the predictive utility of some known risk factors. Study design: A cross-sectional design was used to investigate the prevalence and risk factors for tinnitus. Setting: A questionnaire was distributed, reaching out to a large college-aged population. A total of 2258 young adults aged 18–30 years were recruited from April 2021 to February 2022. Interventions: A questionnaire was administered to investigate the epidemiology of tinnitus in a population of college-aged young adults. Results: About 17.7% of young adults reported bothersome tinnitus perception lasting for ≥5 min in the last 12 months. The prevalence of chronic tinnitus (bothersome tinnitus for ≥1 year) and acute tinnitus (bothersome tinnitus for <1 year) was 10.6% and 7.1%, respectively. About 19% of the study sample reported at least one health condition. Individuals reporting head injury, hypertension, heart disease, scarlet fever, and malaria showed significantly higher odds of reporting chronic tinnitus. Meningitis and self-reported hearing loss showed significant associations with bothersome tinnitus. The prevalence of chronic tinnitus was significantly higher in males reporting high noise exposure, a positive history of reoccurring ear infections, European ethnic background, and a positive health history. Risk modeling showed that noise exposure was the most important risk factor for chronic tinnitus, followed by sex, reoccurring ear infections, and a history of any health condition. A positive history of COVID-19 and self-reported severity showed no association with tinnitus. Individuals reporting reoccurring ear infections showed a significantly higher prevalence of COVID-19. Conclusions: While young adults with health conditions are at a higher risk of reporting tinnitus, the predictive utility of a positive health history remains relatively low, possibly due to weak associations between health conditions and tinnitus. Noise, male sex, reoccurring ear infections, European ethnicity, and a positive health history revealed higher odds of reporting chronic tinnitus than their counterparts. These risk factors collectively explained about 16% variability in chronic tinnitus, which highlights the need for identifying other risk factors for chronic tinnitus in young adults.

## 1. Introduction

Tinnitus, a phantom perception of sound without any external sound source, is a prevalent hearing health condition affecting over fifty million Americans [1,2]. About 15% of individuals exposed to occupational noise and toxic chemicals experience bothersome tinnitus [3,4]. Tinnitus can lead to anxiety, stress, depression, insomnia, cognitive dysfunction, and social isolation, compromising the quality of life of individuals suffering from this condition [5,6,7,8,9,10,11].

Bothersome chronic tinnitus is common among youth, with a prevalence of about 5% in teens and 8% in young adults aged 18–30 years [12,13,14]. The prevalence of bothersome chronic tinnitus is high in children and young adults, and yet, tinnitus in youth remains under-investigated for several reasons. First, young adults can describe tinnitus perception when questioned, but most do not proactively complain about it as they consider it a “normal” phenomenon [14]. Second, about 90% of young adults with tinnitus do not report substantial tinnitus-related distress, an important catalyst that leads tinnitus patients to seek professional help [12]. Third, subjective tinnitus without clinically significant hearing loss in young adults is rarely considered a pathological occurrence that warrants clinical attention [14]. However, emerging evidence suggests that some young adults with bothersome tinnitus might experience subclinical anxiety, depression, and sleep disturbances [6,13]. Young adults reporting bothersome tinnitus perception might be at higher risk of developing clinical manifestations (i.e., tinnitus disorder) at older ages. Accurate risk assessment, individualized counseling, and lifestyle modifications at young ages could help prevent tinnitus disorder. Therefore, there is a need to investigate tinnitus epidemiology in children and young adults.

Recreational noise and music exposure are major risk factors for tinnitus in youth [15,16]. About 90% of college-aged young adults listen to music daily, with 48% reporting their typical volume at maximum or near-maximum levels [17,18,19]. About 44% of young adults are exposed to noisy equipment without using hearing protection [20]. Exposure to firearms and other impulse noises is common for young adults [12]. Attitudes about hearing protection continue to skew negatively in young adults, with concerns about social perception trumping their risk of hearing loss and tinnitus [21]. Acute exposure to loud sound levels could induce cochlear synaptic degeneration without causing permanent hearing loss and contribute to tinnitus perception in young adults with normal hearing sensitivity [22,23,24,25,26]. While noise exposure is a known risk factor for tinnitus, its predictive utility in risk assessment of tinnitus in youth remains under-investigated.

Tinnitus in middle and older adults is often accompanied by health conditions, such as cardiovascular diseases, diabetes, psychiatric disorders, rheumatic diseases, renal conditions, and thyroid gland diseases [27,28,29,30,31]. About 34% of tinnitus patients seeking professional help for bothersome tinnitus reported a positive history of health conditions [31]. Around 47% of tinnitus patients with a positive history of health conditions suffered from hypertension, 34% reported hypercholesterolemia, 22% reported rheumatic diseases, and 16% suffered from diabetes. Tinnitus is associated with ototoxic drugs used to treat medical conditions, such as diuretics, analgesics, antibiotics, anti-inflammatories, corticosteroids, and immunosuppressive medications [29,32]. However, health-related comorbidities associated with tinnitus in young adults remain elusive. Past epidemiological studies investigating tinnitus in youth reported no significant association between tinnitus and physical health conditions [12,13,33,34]. Tinnitus was associated with subclinical manifestations of mental health conditions in youth [6,13], but the association between clinical manifestations of mental health conditions and tinnitus remains unclear. Besides, recent reports suggested an association between audio-vestibular pathologies and COVID-19 [35,36,37,38,39,40]. However, there is a lack of information regarding COVID-19 and tinnitus perception in young adults.

The present study investigated the epidemic of tinnitus in college-aged young adults. We studied the prevalence of tinnitus in a sample of 2258 young adults aged 18–30 years. Our first objective was to identify self-reported health conditions associated with tinnitus in young adults. The second objective was to evaluate their predictive utility in identifying individuals at risk of tinnitus.

## 2. Materials and Methods

### 2.1. Participants

The Institutional Review Board approved the protocol for the present study. Young adults were invited to participate in the present study via university-wide recruitment emails (April 2021–February 2022). We also sent recruitment emails to class instructors and requested them to share the questionnaire link with their students. A sample of 2258 healthy young adults aged 18–30 years were recruited.

### 2.2. Study Questionnaire

The participants filled out a questionnaire that included brief assessments of four areas as follows: demographic details, tinnitus, overall health, and hearing health.

(1) Demographic details: The survey inquired about age, sex, and ethnicity. Ethnicity was reclassified into two categories for statistical purposes: European American and non-European American.

(2) Tinnitus: We adopted questions from the National Health and Nutrition Examination Survey (NHANES). The NHANES database is widely used for reporting tinnitus prevalence in children and young adults [13,14,41]. Tinnitus experience was estimated by asking, “Q.1A. In the past 12 months, have you been bothered by ringing, roaring, buzzing, or any other type of sounds in your ears or head that lasts for 5 min or more?”. Participants who responded with “Yes” to this question were asked the following question about the duration of their tinnitus experiences: “Q.1B. How long have you been bothered by this ringing, roaring, buzzing, or any other sounds in ears or head?” The option choices included “<3 months”, “3–6 months”, “1 to 4 years”, “5–9 years”, or “10 years or more” (see Table S1 in Supplementary Materials). Tinnitus experience was further investigated by asking the following questions: “Q.2. Have you ever experienced ringing, roaring, buzzing, or any other types of sounds in your ears/head?” and “Q.3. Do you hear continuous ringing, roaring, buzzing, or any other types of sounds in your ear or head in quiet situations when you pay attention?”. No tinnitus was defined as no tinnitus experience (“No” to Q.1A and Q.2). Individuals who responded positively to Q.1A were categorized into two groups: chronic tinnitus and acute tinnitus. Acute tinnitus and chronic tinnitus were defined as tinnitus experienced for <1 year and ≥1 year, respectively. Individuals with no experience of bothersome tinnitus (“No” to Q.1A and “Yes” to Q.2) were defined as subacute tinnitus. Continuous tinnitus and intermittent tinnitus were characterized as “Yes” and “No” responses to Q.3, respectively.

(3) Medical health history: The survey asked the following question about past illnesses: “What illnesses do you have, or have you had?” The answer choices included “meningitis”, “head injury”, “heart disease”, “scarlet fever”, “diabetes”, and “hypertension”. Participants were asked to list any other physical or mental health conditions not listed in the options. Health history was classified into two categories for statistical analysis: positive and negative health history. We inquired about COVID-19 infection by asking, “Have you ever been infected with COVID-19?”. If the participants responded affirmatively, a follow-up question was asked about the perceived level of COVID-19 severity: “Please indicate the overall symptom severity while you were infected”. The response choices included “Very mild”, “Mild”, “Moderate”, “Severe”, and “Very severe”.

(4) Reoccurring ear infection and noise exposure: We adopted the NHANES questions to investigate reoccurring ear infections and noise exposure. The survey inquired about reoccurring ear infections by asking, “Have you ever had three or more ear infections?”. The response choices included “Yes” and “No”. Noise exposure was inquired by asking, “Have you been exposed to loud recreational or occupational noise/music for more than 10 h a week over the past year?”. The response choices included “Never”, “Sometimes”, “About half the time”, “Most of the time”, and “Always”.

### 2.3. Statistical Analyses

Statistical analyses were conducted using SPSS software version 27 (IBM Corp., Armonk, NY, USA). We used multinomial logistic regression analysis to identify risk factors for chronic, acute, subacute, and any form of tinnitus. The regression model used the following independent variables—age, sex, ethnicity, reoccurring ear infections, health history, noise exposure, and history of COVID-19. Odds ratio (OR) and 95% confidence intervals were obtained. Pearson’s product–moment correlation coefficients and Chi-square statistics were calculated to investigate the relationship between the experimental variables.

We employed risk modeling in RStudio (R-Tools Technology, Inc, Boston, MA, USA) using a categorical tinnitus variable with four levels—chronic tinnitus, acute tinnitus, subacute tinnitus, and no tinnitus. To investigate the predictive utility of the response variables, we constructed risk assessment models with logistic regression [42], support vector machine [43], random forest [44], K-nearest neighbors classifier [45], Gaussian naive Bayes [46], and extremely gradient boosted decision trees [47]. To control overfitting and to evaluate the generalizability of the candidate machine learning models, we applied a 3-fold cross-validation procedure [48]. We used the receiver operating characteristic curve (ROC) to visualize the performance of the classification models [49]. We quantified model accuracy by calculating the area under the curve (AUC) [50]. The model with the highest average AUC after 3-fold cross-validation was selected as the best model for further interpretability analysis. After the model selection, we applied Shapley additive explanations (SHAP) to quantify the relative contributions of each predictor variable in the model’s performance [51]. We computed SHAP values to quantify local feature importance scores, which were then averaged to calculate global feature importance scores [51]. The local feature importance scores assist in determining how much that feature has contributed to the prediction of that sample. On the other hand, the global feature importance score helps in better understanding and identification of important features, as well as interactions between various features.

## 3. Results

### 3.1. Demographic Details of the Study Sample

**Figure 1:**
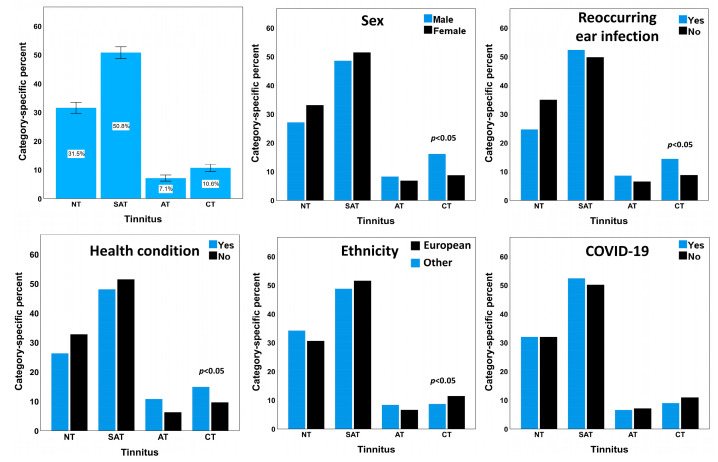
A bar chart on the top left panel shows the prevalence of tinnitus in the study sample. The rest of the bar charts show the category-specific prevalence of tinnitus for sex, ethnicity, reoccurring ear infections, any health conditions, and COVID-19.

Table 1 provides the demographic details of the study sample. The study sample included data from 2258 young adults aged 18–30 years. Among the study sample, 240 participants (10.6%) reported chronic tinnitus and 160 (7.1%) participants reported acute tinnitus (Figure 1). About 19% of the participants reported having at least one health condition. The prevalence of head injury, meningitis, hypertension, and diabetes in the study sample was about 9.8%, 0.4%, 1.5%, and 0.4%, respectively. The prevalence of heart diseases, malaria, and scarlet fever was about 1.7%, 0.7%, and 0.6%, respectively. About 57.3% of the participants reported exposure to loud recreational or occupational noise/music for more than 10 h a week over the past year. About 23% of the participants reported a positive history of COVID-19 infection.

### 3.2. Health Conditions Associated with Tinnitus

**Figure 2:**
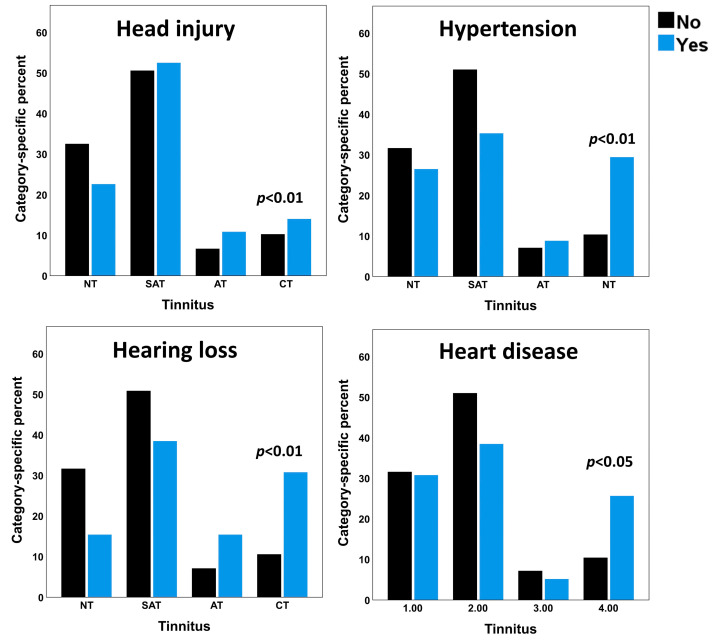
Bar charts showing the category-specific prevalence of tinnitus between individuals with and without a positive history of health conditions. Hypertension, head injury, hearing loss, and heart disease revealed a significant association with tinnitus.

Among the study sample, 437 individuals (19.3%) reported a positive history of health conditions (excluding COVID-19). Among those, 221 (50.6%) reported head injury, 39 (8.9%) reported heart disease, 16 (3.7) reported mumps, 15 (3.4%) reported malaria, 14 (3.2%) reported scarlet fever, 10 (2.3%) reported diabetes, 9 (2.1%) reported meningitis and 150 (34.3%) reported more than one or other health conditions. Appendix A presents the frequency and row percentage of tinnitus categories for an exhaustive list of health conditions in the sample. Chi-squares were calculated for health conditions with at least five individuals in a single cell of 2 × 2 contingency tables between chronic tinnitus and health conditions. Head injury (*X*^2^ (1, *N* = 952) = 8.01, *p* = 0.005), hypertension (*X*^2^ (1, *N* = 952) = 7.73, *p* = 0.005), heart disease (*X*^2^ (1, *N* = 952) = 4.89, *p* = 0.027), scarlet fever (*X*^2^ (1, *N* = 952) = 5.95, *p* = 0.01), malaria (*X*^2^ (1, *N* = 952) = 4.43, *p* = 0.035), and a history of any other conditions (*X*^2^ (1, *N* = 952) = 7.37, *p* = 0.007) showed significant associations with chronic tinnitus (Figure 2). We performed Chi-square analyses between health conditions and bothersome tinnitus (i.e., acute tinnitus and chronic tinnitus combined). In addition to the health conditions associated with chronic tinnitus, meningitis (*X*^2^ (1, *N* = 2258) = 8.87, *p* = 0.003) and self-reported hearing loss (*X*^2^ (1, *N* = 2258) = 7.25, *p* = 0.007) showed significant association with bothersome tinnitus (Table 2, Figure 2).

### 3.3. Results of the Regression Analyses

Table 3 presents the results of the multinomial regression analyses. The regression models compared the prevalence of no tinnitus and a specific tinnitus category between the levels of the dependent variables. Individuals with high noise exposure, positive history of reoccurring ear infections, and positive health history showed significantly higher odds of reporting acute tinnitus (see Figure S2 in Supplementary Materials). The regression model explained about 10% of the variance (Cox and Snell pseudo-R-square = 0.1, *p* < 0.05) in the acute tinnitus measure. The prevalence of chronic tinnitus was higher in males reporting high noise exposure, positive history of reoccurring ear infections, European ethnic background, and positive health history than their counterparts. The regression model explained about 16% of the variance in chronic tinnitus (Cox and Snell pseudo-R-square = 0.16, *p* < 0.05, Figure 1). Continuous tinnitus was associated with sex, health history, noise exposure, and reoccurring ear infections (see Appendix B and Figure S2 from Supplementary Materials). COVID-19 showed no association with any tinnitus category (see Figure S3 in Supplementary Materials).

### 3.4. Association between COVID-19 and Reoccurring Middle Ear Infections

Individuals with a positive history of COVID-19 showed a significantly higher prevalence of reoccurring ear infections (*X*^2^ (1, *N* = 2258) = 12.06, *p* < 0.001) (see Figure S1 in Supplementary Materials). We obtained a significant Chi-square result for COVID-19 severity and reoccurring ear infections (*X*^2^ (4, *N* = 2258) = 22.68, *p* < 0.001). A post hoc analysis showed (adjusted *p* = 0.005) that the prevalence of reoccurring ear infections was significantly higher in participants reporting mild and moderate levels of COVID-19 severity. The effect of reoccurring middle ear infections remained significant even after controlling for ethnicity, sex, noise exposure, and age using the logistic regression model (OR = 1.40, *p* = 0.004).

### 3.5. Risk Modeling for Tinnitus

**Figure 3:**
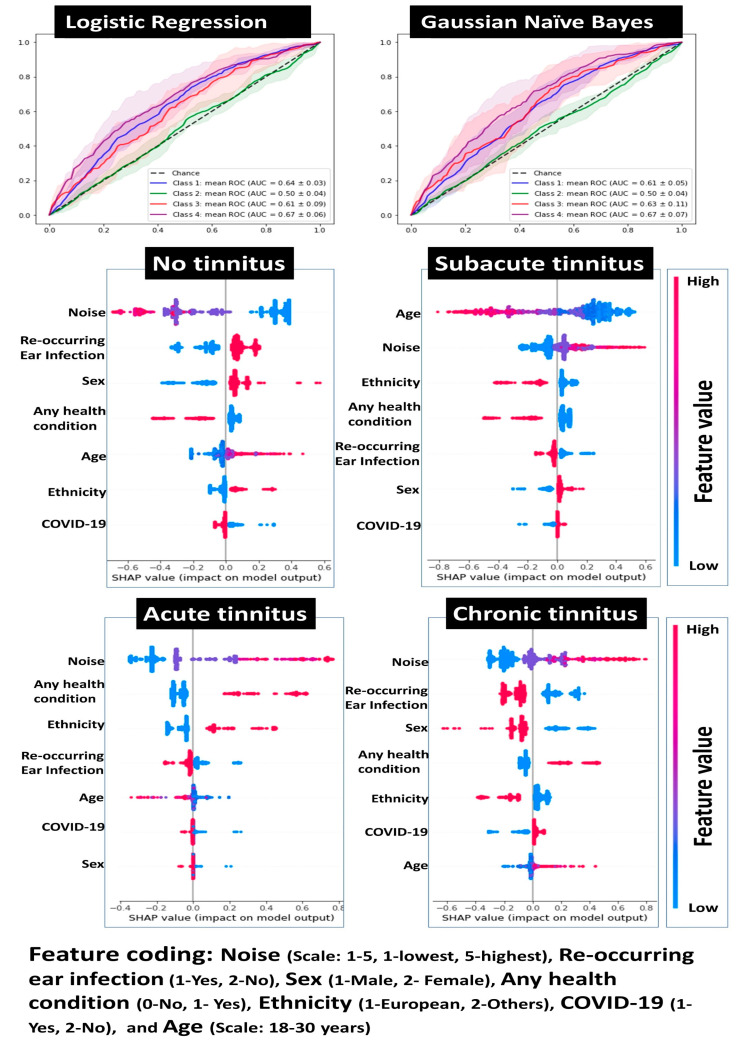
The top row presents the receiver operating curves (ROC) (mean ± 95% CI) for tinnitus categories obtained using the logistic regression model and Gaussian Naive Bayes. The logistic regression model revealed the highest ROC values for predicting tinnitus categories among other tested models. The middle and bottom rows present tinnitus category-specific feature analysis for the logistic regression model. The Shapley additive explanations (SHAP) values present the relative importance of the predictors. Positive SHAP values for a specific feature indicate that the model weighted it higher than the features with negative SHAP values. For example, the model weight noise exposure values “4” and “5” were more than “1” and “2” while categorizing a participant in the chronic tinnitus group. The model weighted noise exposure values “1” and “2” were more than “4” and “5” while categorizing a participant in the no tinnitus group. The SHAP values for each model are sorted in descending order of importance of the predictors contributing to the model’s performance.

Figure 3 presents the performance of the top risk assessment models. The logistic regression model was chosen as the best fit after the three-fold cross-validation. Chronic tinnitus and no tinnitus categories achieved AUC values of 0.67 (*p* < 0.05) and 0.64 (*p* < 0.05), respectively. The SHAP values showed that the model weighted high noise exposure more than a positive history of reoccurring ear infections, male sex, a positive history of any health conditions, and European ethnicity for predicting chronic tinnitus. The model weighted low noise exposure more than no history of reoccurring ear infections, female sex, and no history of any health conditions for no tinnitus. Noise exposure emerged as the most important risk factor for chronic tinnitus, followed by reoccurring ear infections, sex, history of any health condition, and ethnicity.

## 4. Discussion

The major findings of the present study were as follows: (1) In a sample of 2258 young adults, the prevalence of chronic, acute, and subacute tinnitus was around 10.6%, 7.1%, and 50.8%, respectively. (2) Head injury, hypertension, heart disease, scarlet fever, malaria, and a history of any other health conditions were associated with chronic tinnitus. Meningitis and self-reported hearing loss showed a significant association with bothersome tinnitus. (3) Exposure to loud recreational or occupational noise/music for more than 10 h/week was identified as the single most important significant risk factor for chronic tinnitus in young adults. (4) Males showed a significantly higher prevalence of chronic tinnitus compared to females. (5) COVID-19 and reoccurring ear infections showed a significant association, while COVID-19 showed no association with tinnitus. The association between tinnitus and risk factors such as meningitis, malaria, and scarlet fever must be interpreted with caution because only a small number of participants in the current sample reported these conditions.

### 4.1. Prevalence of Tinnitus in Youth

The prevalence of tinnitus in children and teens with normal hearing ranges from 4.7 to 46% rises to 23.5–62.2% in children and teens with hearing loss [52]. The prevalence of bothersome tinnitus in children and young adults ranges from 4.7 to 23.2% [12,13,53,54]. The prevalence is critically dependent on methodological factors, such as the study population, sampling procedures, operational definitions, tinnitus questions, and contextual factors. There are no widely accepted screening criteria for evaluating tinnitus. A wide range of screening criteria is employed by epidemiological studies [55], but no comparative analyses have been performed on their accuracy. We utilized the NHANES questions for evaluating tinnitus to facilitate comparison across nationally representative studies [1,5,6,13,41,56,57,58,59,60,61]. Healthy young adults with chronic tinnitus manifest a mild form of subclinical tinnitus, and they might be at higher risk of tinnitus disorder at older ages. Our results reiterated that bothersome tinnitus is a prevalent hearing health condition among young adults. Future effects should be directed to prevent tinnitus disorder among young adults with chronic tinnitus.

### 4.2. Health-Related Factors Associated with Tinnitus in Young Adults

Head injury is a known risk factor for tinnitus in older adults aged 41 years and above [14,62,63,64]. A past study suggested that an abnormal increase in the gain of the cochlear amplifier followed by a reduction in central efferent suppression might contribute to head injury-related tinnitus [65]. Patients with head injuries often experience more tinnitus-related distress than their counterparts [66,67]. The exact neurophysiological mechanisms underlying head injury-induced tinnitus remain elusive, suggesting the need for more research [64].

Cardiovascular conditions are known risk factors for tinnitus in older adults [1,59,62,68]. Past studies could not observe the relationship between tinnitus and cardiovascular conditions in young adults [12,13]. We obtained a significant association between tinnitus and hypertension in young adults. These findings are consistent with a recent study using the NHANES database that showed a significant association between bothersome tinnitus and hypertension in young adults [69]. We observed significant tinnitus associations with scarlet fever, malaria, and meningitis, but these associations should be interpreted with caution because only a small number of participants reported these conditions. Scarlet fever is caused by infective Group A Streptococcal bacteria [70]. Tinnitus and acquired hearing loss have been observed in patients with scarlet fever [71,72,73]. Quinoline, a drug used for treating malaria, has documented ototoxic properties and is associated with tinnitus [74]. The association between tinnitus and meningitis was observed for older adults in the Blue Mountains Hearing Study cohort [75]. Further research is needed to investigate the mechanisms underlying the observed association in the present study.

### 4.3. Noise as a Major Risk Factor for Tinnitus in Young Adults

Noise exposure was identified as the most important risk factor for tinnitus in young adults. Recent reports suggest that about 1.1 billion young adults are at risk of permanently damaging their auditory system due to recreational noise exposure [76]. Up to 58% of adults using personal music listening devices could exceed the NIOSH recommended noise dose [77]. About 86% of young adults report that they do not receive guidelines or recommendations for hearing protection before exposure to loud noise or music [77,78,79]. In clinics, the NIOSH noise damage risk criteria are used to determine the permissible noise dose, categorize individuals based on noise exposure, and provide counseling for preventing tinnitus and noise-induced hearing loss [80,81,82,83]. The NIOSH standards are designed to prevent permanent threshold shifts in factory workers routinely exposed to occupational noise over 40 years of work life and do not account for the risk of tinnitus [80]. There is an increasing concern that the noise exposure standards are inadequate to prevent noise-induced damage to the ribbon synapses between the inner hair cells and afferent auditory nerve fibers, which might contribute to tinnitus [84,85]. The NIOSH standards designed for preventing permanent threshold shifts in factory workers might not be equally efficient for preventing bothersome chronic tinnitus in young adults, highlighting the need for designing novel strategies for tinnitus prevention in young adults.

### 4.4. Demographic and Health-Related Risk Factors of Tinnitus in Young Adults

The present study found a higher prevalence of chronic tinnitus in individuals reporting European ethnic backgrounds (Table 1), which is consistent with other reports [1,5,12,13]. The prevalence of chronic tinnitus and any form of tinnitus was higher in males than in females (Table 1). These findings are consistent with past studies investigating tinnitus in older adults [5,86,87]. However, other studies investigating young adults have reported opposite findings, with females showing a higher prevalence of chronic tinnitus and noise-induced hearing loss than males [13,88]. Bhatt [12] reported a higher prevalence of acute tinnitus and any form of tinnitus in females than in males, which was not replicated in the present study (Table 1). The methodological factors, such as survey distribution and ethnic differences between the samples, might contribute to the differences in the findings, suggesting the need for further investigations.

### 4.5. Reoccurring Ear Infections as a Risk Factor for Tinnitus

The reoccurring ear infection was significantly associated with tinnitus categories, which is consistent with past studies [12,13,62,89,90,91]. Reoccurring ear infections could cause cochlear deafferentation [92]. Oral antibiotics are often prescribed for otitis media with effusion, although oral antibiotic treatment is not recommended by the clinical practice guidelines endorsed by the American Academy of Otolaryngology—Head and Neck Surgery Foundation and the American Academy of Pediatrics [93]. Ototopical aminoglycosides with known ototoxic effects are frequently used to treat suppurative otitis media [94]. Cochlear deafferentation due to reoccurring ear infections and exposure to ototoxic therapeutics might be putative mechanisms underlying the association. Further research is needed to investigate the effects of reoccurring ear infections on tinnitus.

### 4.6. Tinnitus and COVID-19

COVID-19 and self-reported severity were not associated with any form of tinnitus in young adults (See Figure S3 in Supplementary Materials). Most studies investigated tinnitus and COVID-19 in older adults with age-related confounding factors, such as pre-existing health conditions [35,36,95]. It remains unclear if the association between COVID-19 and tinnitus in past studies could be attributed to COVID-19, pre-existing health conditions, or therapeutics used for treatment. We could not obtain a significant relationship between tinnitus and COVID-19 in young adults in the present study. The prevalence of “very severe” symptoms was 0%, and about 91% of young adults had mild COVID-19 symptoms. A large-scale investigation is necessary to examine the effects of COVID-19 on tinnitus in young adults.

### 4.7. Reoccurring Ear Infection and COVID-19

COVID-19 and reoccurring ear infections showed a significant association. The mechanisms underlying this relationship remain elusive. A shared genetic component to the susceptibility to infectious agents and COVID-19 could explain the observed association; however, the genetic susceptibility to reoccurring infection is not studied well [96]. Health-related habits (e.g., hygiene) [97], environmental factors, and health-related disparities might mediate their effects on reoccurring ear infections and COVID-19. Further research is needed to investigate the relationship between reoccurring ear infections and COVID-19.

### 4.8. Association between Mental Health Conditions and Tinnitus in Young Adults

Tinnitus is associated with anxiety, depression, sleep disturbances, and other mental health conditions [98]. Recent studies identified shared genetic underpinnings between tinnitus and mental health traits [99,100]. Young adults with subclinical manifestations of tinnitus might experience mental health issues [13]. However, the present study found that only seven participants (one with NT, five with SAT, and one with CT) reported mental health conditions, which prevented us from performing inferential statistics. Mental health conditions are underreported and stigmatized in young adults [101]. In addition, it is possible that young adults with tinnitus experience subclinical mental health problems without being consciously aware of their distress. A comprehensive test battery sensitive to subclinical mental distress is necessary to elucidate the relationship between tinnitus and mental health conditions in young adults.

### 4.9. Experimental Caveats

Our cross-sectional study utilized a brief questionnaire to investigate the tinnitus epidemic in young adults. This study did not evaluate an exhaustive list of risk factors for tinnitus (such as organic solvents or recreational drugs and co-existing subclinical mental health conditions) and was dependent on self-reported health conditions. We could not rule out a participation bias, which might motivate some participants to complete the study questionnaire. Furthermore, no audiological or psychological measures were obtained in the current study. The inclusion of psychological measures might produce stronger associations than those reported in the current study.

## 5. Conclusions

The prevalence of chronic tinnitus was 10.6% in young adults aged 18–30 years. Exposure to loud recreational or occupational noise/music for more than 10 h/week is a leading risk factor for tinnitus. Males with noise exposure, reoccurring ear infections, European ethnicity, and a positive health history revealed higher odds of reporting chronic tinnitus than their counterparts. These risk factors collectively explained about 16% variability in chronic tinnitus, highlighting the need for further research.

## Figures and Tables

**Table 1:** Demographic and health-related factors of young adults aged 18–30 years with no tinnitus (NT), sub-acute tinnitus (ST), acute tinnitus (AT), or chronic tinnitus (CT) (*N* = 2258) displayed using frequency and row section percentage.

Variables	Tinnitus (Frequency (%))			
	NT	ST	AT	CT
Sex				
Male	148 (6.7)	265 (11.7)	45 (2.0)	88 (3.9)
Female	559 (24.7)	868 (38.5)	115 (5.1)	147 (6.5)
Non-binary	3 (0.1)	7 (0.3)	0 (0)	5 (0.2)
No disclosure	2 (0.08)	6 (0.2)	0 (0)	0 (0)
Ethnicity				
Europeans	503 (22.3)	848 (37.6)	109 (4.8)	187 (8.3)
Others	209 (9.3)	298 (13.2)	51 (2.3)	53 (2.3)
Reoccurring ear infections				
Yes	152 (6.7)	323 (14.3)	53 (2.3)	89 (3.9)
No	560 (24.8)	823 (36.4)	107 (4.7)	151 (6.7)
Health history				
Present	115 (5.1)	210 (9.3)	47 (2.1)	65 (2.9)
Absent	597 (26.4)	936 (41.5)	113 (5.0)	175 (7.8)
Noise				
Always	7 (0.3)	24 (1.1)	11 (0.5)	10 (0.5)
Most of the time	18 (0.8)	79 (3.7)	13 (0.6)	28 (1.3)
About half the time	35 (1.7)	81 (3.8)	26 (1.2)	29 (1.4)
Sometimes	197 (9.3)	441 (20.8)	57 (2.7)	100 (4.7)
Never	405 (19.1)	456 (21.5)	45 (2.1)	59 (2.8)
COVID-19				
Yes	164 (7.3)	269 (11.9)	47 (2.1)	65 (2.9)
No	548 (24.3)	877 (38.8)	126 (5.6)	194 (8.6)

**Table 2:** Frequency and row percentage along with Chi-square analysis of seven major health conditions among participants with no tinnitus, subacute tinnitus, acute tinnitus, and chronic tinnitus.

		No Tinnitus	Subacute Tinnitus	Acute Tinnitus	Chronic Tinnitus	Chi-Square	*p*-Value
		Count (Row %)	Count (Row %)	Count (Row %)	Count Row %		
Head injury	No	662 (32.5)	1030 (50.6)	136 (6.7)	209 (10.3)	8.01	0.005
	Yes	50 (22.6)	116 (52.5)	24 (10.9)	31 (14.0)		
Hypertension	No	703 (31.6)	1134 (51.0)	157 (7.1)	230 (10.3)	7.73	0.005
	Yes	9 (26.5)	12 (35.3)	3 (8.8)	10 (29.4)		
Hearing loss	No	710 (31.6)	1141 (50.8)	158 (7)	236 (10.5)	7.25	0.007
	Yes	2 (15.4)	5 (38.5)	2 (15.4)	4 (30.8)		
Heart disease	No	700 (31.5)	1131 (51.0)	158 (7.1)	230 (10.4)	4.89	0.027
	Yes	12 (30.8)	15 (38.5)	2 (5.1)	10 (25.6)		
Malaria	No	708 (31.6)	1143 (51.0)	157 (7.0)	235 (10.5)	4.43	0.035
	Yes	4 (26.7)	3 (20.0)	3 (20.0)	5 (33.3)		
Scarlet fever	No	709 (31.6)	1141 (50.8)	159 (7.1)	235 (10.5)	5.95	0.01
	Yes	3 (21.4)	5 (35.7)	1 (7.1)	5 (35.7)		
Any health condition	No	597 (32.8)	936 (51.4)	113 (6.2)	175 (9.6)	7.37	0.007
	Yes	115 (26.3)	210 (48.1)	47 (10.8)	65 (14.9)		

Chi-square was performed on a 2 × 2 contingency table between chronic tinnitus (two categories: chronic tinnitus versus other categories) and health condition (two categories: yes to health condition versus no to health condition).

**Table 3:** Results of the multinomial regression analyses. The odds ratio and 95% of the confidence interval are presented. Model-specific pseudo R^2^ values are reported.

Variables	Subacute Tinnitus	Acute Tinnitus	Chronic Tinnitus	Any Tinnitus
Pseudo R-square (Cox and Snell)	0.04	0.10	0.16	0.05
Age (covariate)	0.96 (0.92–1.01)	0.97 (0.93–0.99) *	1.02 (0.96–1.08)	0.97 (0.93–1.00)
Sex				
Male	1.23 (0.98–1.58)	1.37 (0.89–2.09)	2.26 (1.58–3.24) **	1.37 (1.08–1.73) *
Female	1	1	1	1
Ethnicity				
Europeans	1.10 (0.87–1.38)	0.81 (0.54–1.21)	1.58 (1.07–2.34) *	1.11 (0.89–1.39)
Non-Europeans	1	1	1	1
Reoccurring ear infections				
Yes	1.40 (1.11–1.77) *	1.66 (1.11–2.47) *	2.28 (1.61–3.23) **	1.53 (1.22–1.91) **
No	1	1	1	1
Any health condition				
Present	1.11 (0.85–1.45)	1.94 (1.28–2.95) *	1.58 (1.08–2.32) *	1.25 (0.97–1.61)
Absent	1	1	1	1
Noise exposure				
Always	2.72 (1.15–6.44) *	12.86 (4.69–35.24) ***	8.22 (2.95–22.89) **	4.10 (1.82–9.25) **
Most of the time	3.91 (2.26–6.74) ***	6.10 (2.77–13.49) ***	10.9 (5.53–21.5) ***	4.75 (2.80–8.06) ***
About half the time	1.75 (1.14–2.68) *	5.71 (3.12–10.46) ***	4.87 (2.71–8.76) ***	2.38 (1.59–3.55) **
Sometimes	1.90 (1.52–2.36) ***	2.43 (1.58–3.75) **	3.51 (2.40–5.14) ***	2.09 (1.70–2.59) ***
Never	1	1	1	1
COVID-19				
Yes	0.95 (0.75–1.20)	0.81 (0.52–1.27)	0.73 (0.49–1.09)	0.91 (0.72–1.14)
No	1	1	1	1

* *p* < 0.05; ** *p* < 0.001; *** *p* < 10^−5^.

## Data Availability

The data presented in this study are not publicly available due to ethical constraints but are available upon request from the corresponding authors.

## Supplementary Materials

The following supporting information can be downloaded at: https://www.mdpi.com/article/10.3390/audiolres13040048/s1, Figure S1: A bar chart showing the prevalence of COVID-19 between individuals with and without a history of reoccurring ear infections. Individuals with reoccurring ear infections reported a significantly higher prevalence of COVID-19; Figure S2: A bar chart showing the noise category-specific prevalence of tinnitus. The prevalence of chronic tinnitus rises from about 6% to 20% as a function of the noise exposure category, showing that chronic tinnitus is significantly associated with noise exposure; Figure S3: A bar chart showing the COVID-19 severity-specific percentage among tinnitus categories, suggesting no significant association between COVID-19 and tinnitus category in young adults; Table S1: Study Questionnaire.

## Appendix A

**Table A1:** Frequency and row percentage of health conditions among participants with no tinnitus, subacute tinnitus, acute tinnitus, and chronic tinnitus.

		No Tinnitus		Subacute Tinnitus		Acute Tinnitus		Chronic Tinnitus	
		Count	Row %	Count	Row %	Count	Row %	Count	Row %
Meningitis ^++^	No	709	31.5	1145	50.9	159	7.1	236	10.5
	Yes	3	33.3	1	11.1	1	11.1	4	44.4
Hearing loss ^+++^	No	710	31.6	1141	50.8	158	7.0	236	10.5
	Yes	2	15.4	5	38.5	2	15.4	4	30.8
Hypertension **	No	703	31.6	1134	51.0	157	7.1	230	10.3
	Yes	9	26.5	12	35.3	3	8.8	10	29.4
Low blood pressure	No	712	31.6	1143	50.7	160	7.1	240	10.6
	Yes	0	0.0	3	100.0	0	0.0	0	0.0
Head injury **	No	662	32.5	1030	50.6	136	6.7	209	10.3
	Yes	50	22.6	116	52.5	24	10.9	31	14.0
Heart disease *	No	700	31.5	1131	51.0	158	7.1	230	10.4
	Yes	12	30.8	15	38.5	2	5.1	10	25.6
Malaria *	No	708	31.6	1143	51.0	157	7.0	235	10.5
	Yes	4	26.7	3	20.0	3	20.0	5	33.3
Scarlet fever *	No	709	31.6	1141	50.8	159	7.1	235	10.5
	Yes	3	21.4	5	35.7	1	7.1	5	35.7
Diabetes	No	707	31.5	1144	50.9	160	7.1	237	10.5
	Yes	5	50.0	2	20.0	0	0.0	3	30.0
Mumps	No	708	31.6	1139	50.8	157	7.0	238	10.6
	Yes	4	25.0	7	43.8	3	18.8	2	12.5
Temporomandibular joint disorder	No	712	31.5	1146	50.8	159	7.0	240	10.6
	Yes	0	0.0	0	0.0	1	100.0	0	0.0
Any health condition **	No	597	32.8	936	51.4	113	6.2	175	9.6
	Yes	115	26.3	210	48.1	47	10.8	65	14.9

** *p* < 0.01 showing comparison between chronic tinnitus and a health condition; * *p* < 0.01 showing comparison between chronic tinnitus and a health condition; ^+++^ *p* < 0.001 showing comparison between chronic tinnitus and a health condition; ^++^ *p* < 0.01 showing comparison between chronic tinnitus and a health condition.

## Appendix B

**Table A2:** Results of the multinomial regression analyses for continuous and intermittent tinnitus.

Variables	Intermittent Tinnitus	Continuous Tinnitus
Pseudo R-square (Cox and Snell)	0.05	0.12
Age (covariate)	0.97 (0.94–1.01)	0.96 (0.92–1.01)
Sex		
Male	1.24 (0.94–1.01)	1.63 (1.22–2.17) **
Female	1	1
Ethnicity		
Europeans	1.18 (0.93–1.49)	1.00 (0.76–1.31)
Non-Europeans	1	1
Reoccurring ear infections		
Yes	1.46 (1.15–1.85) *	1.66 (1.26–2.19) **
No	1	1
Health history		
Present	1.17 (0.89–1.53)	1.41 (1.04–1.92) *
Absent	1	1
Noise exposure		
Always	3.14 (1.32–7.45) *	6.25 (2.58–15.11) ***
Most of the time	3.89 (2.23–6.78) ***	6.62 (3.70–11.83) ***
About half the time	1.88 (1.21–2.90) *	3.46 (2.18–5.49) ***
Sometimes	2.00 (1.60–2.51) ***	2.31 (1.76–3.03) ***
Never	1	1
COVID-19		
Yes	1.07 (0.81–1.42)	1.07 (0.81–1.42)
No	1	1

* *p* < 0.05, ** *p* < 0.001, *** *p* < 10^−5^.
