# Supplementary material for: Identifying Health-Related Conditions Associated with Tinnitus in Young Adults
Source: Audiol Res. 2023 Jul 20;13(4):546–62. doi: 10.3390/audiolres13040048 (PMC10366783; doi:10.3390/audiolres13040048)
Supplement: Supplementary file 1 [file audiolres-13-00048-s001.zip › audiolres-2332514-supplementary.pdf]

Supplementary Files

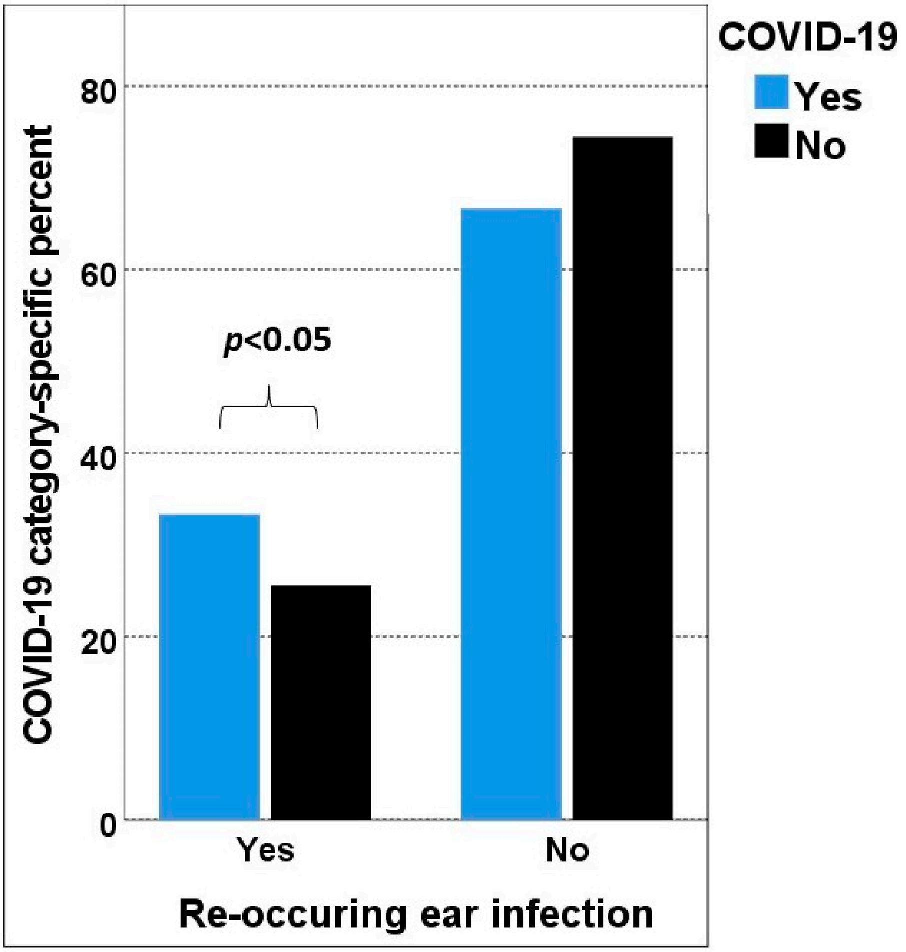

Figure S1: A bar chart showing the prevalence of COVID-19 between individuals with and without a history of reoccurring ear infections. Individuals with reoccurring ear infections reported a significantly higher prevalence of COVID-19 (NT – Not tinnitus, SAT = subacute tinnitus, AT – acute tinnitus, and CT – chronic tinnitus).

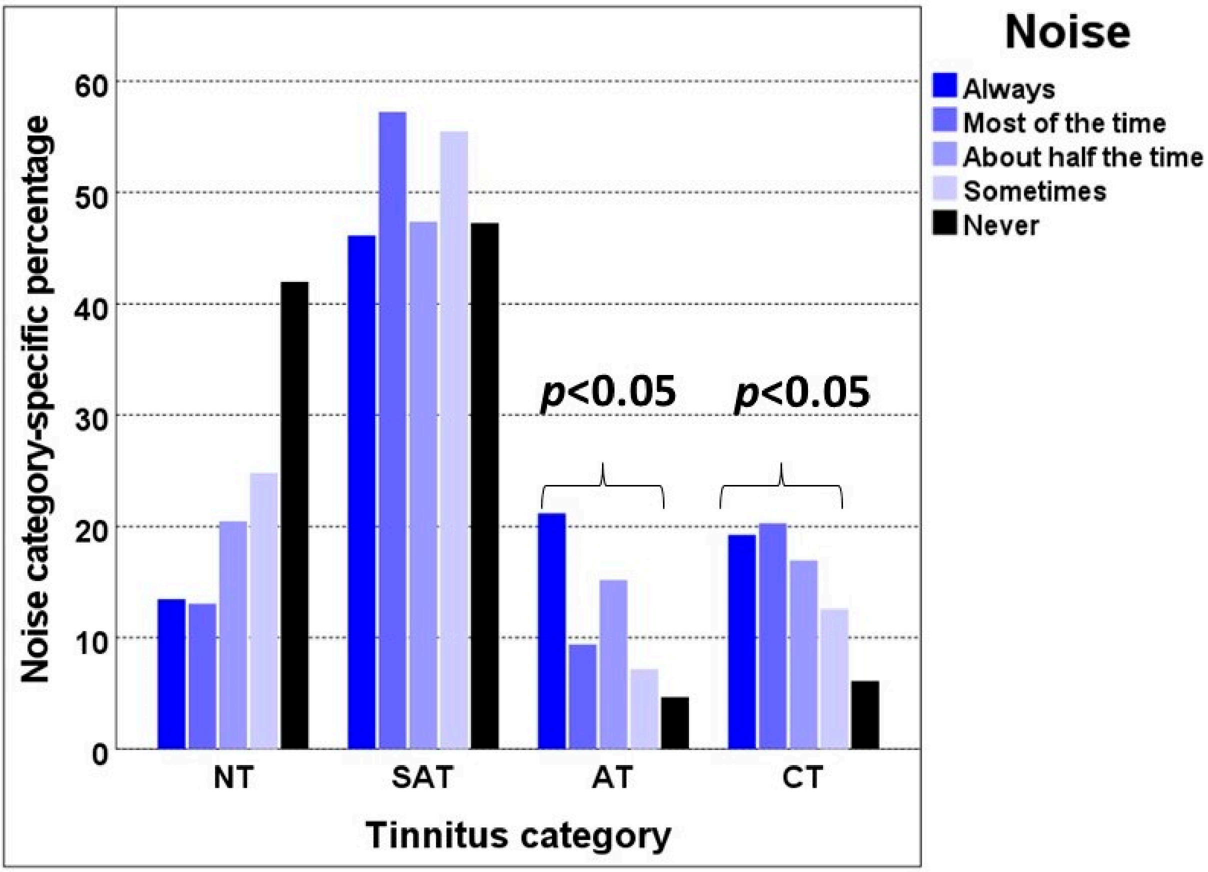

Figure S2: A bar chart showing the noise category-specific prevalence of tinnitus. The prevalence of chronic tinnitus rises from about 6% to 20% as a function of the noise exposure category, showing that chronic tinnitus is significantly associated with noise exposure (NT – Not tinnitus, SAT = subacute tinnitus, AT – acute tinnitus, and CT – chronic tinnitus).

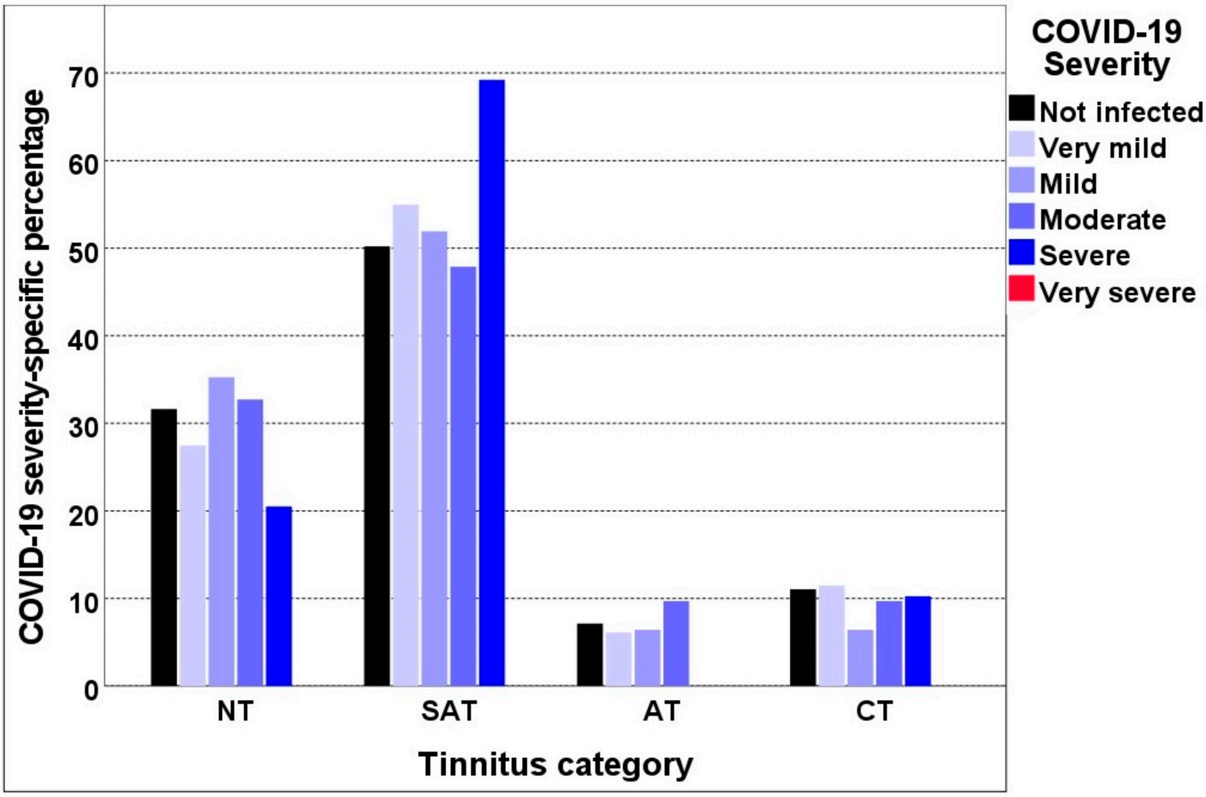

Figure S3: A bar chart showing the COVID-19 severity-specific percentage among tinnitus categories, suggesting no significant association between COVID-19 and tinnitus category in young adults (NT – Not tinnitus, SAT = subacute tinnitus, AT – acute tinnitus, and CT – chronic tinnitus).

Table S1. Study Questionnaire

Study Questionnaire	
Please read all the questions carefully before answering them.	
1	**Demographic Details**
**What is your age?**<br>______ years<br>**What is your gender?**<br>☐Male<br>☐Female<br>☐Other (Please specify):___________<br>☐No disclosure<br>**What is your ethnicity? Please check all that apply.**<br>☐Black/ African American/ Afro Caribbean<br>☐White<br>☐Hispanic/ Latino<br>☐Native Hawaiian/Other Pacific Islander<br>☐American Indian/ Alaskan Native<br>☐ Middle Eastern<br>☐ South Asian<br>☐ East Asian<br>☐ Other ________________	
2	**Tinnitus**
**In the past 12 months, have you been bothered by ringing, roaring, buzzing, or any other type of sounds in ears or head that lasts for 5 minutes or more?**<br>☐Yes ☐No<br>**If you answered "Yes" to the above question, then please answer how long have you been bothered by this ringing, roaring, or buzzing in ears or head?**<br>☐Less than three months<br>☐Three months to a year<br>☐1 to 4 years<br>☐5 to 9 years<br>☐10 or more years<br>☐Don't know<br>**Have you ever experienced ringing, roaring, or buzzing in your ears/head?**<br>☐Yes ☐No<br>**If yes, do you hear continuous ringing, roaring, or buzzing in silence when you pay attention?**<br>☐Yes ☐No	
3	**Overall health**
**What illnesses do you have, or have you had? Please check all that apply.** ☐None<br>Please select all that apply:<br>☐ Meningitis<br>☐ Heart disease<br>☐ High Blood Pressure<br>☐ Head Injury<br>☐ Diabetes<br>☐ Scarlet fever<br><br>**Please list other health conditions that you have or had in the past (including physical and mental health conditions):**<br>____<br>____<br>____<br>______________________________	

**Have you ever been infected with COVID-19?** ☐Yes ☐No<br>**If yes, please indicate overall symptom severity while you were infected:**<br>☐Very mild ☐Mild ☐Moderate ☐Severe ☐Very severe	
**4**	**Hearing health**
**Do you have a history of ear infections?** ☐Yes ☐No<br><br>**Ever had 3 or more ear infections?** ☐Yes ☐No<br><br>**Have you been exposed to loud recreational or occupational noise/music for more than 10 hours a**<br><br>**week over the past year?** ☐Never ☐Sometimes ☐About half the time ☐Most of the time ☐Always	
